# Enhanced Activity of Meprin-α, a Pro-Migratory and Pro-Angiogenic Protease, in Colorectal Cancer

**DOI:** 10.1371/journal.pone.0026450

**Published:** 2011-11-11

**Authors:** Daniel Lottaz, Christoph A. Maurer, Agnès Noël, Silvia Blacher, Maya Huguenin, Alexandra Nievergelt, Verena Niggli, Alexander Kern, Stefan Müller, Frank Seibold, Helmut Friess, Christoph Becker-Pauly, Walter Stöcker, Erwin E. Sterchi

**Affiliations:** 1 Department of Rheumatology, Clinical Immunology and Allergology, Inselspital, University Hospital of Bern, Bern, Switzerland; 2 Institute of Biochemistry and Molecular Medicine, University of Bern, Bern, Switzerland; 3 Department of Surgery, Kantonsspital Liestal, Liestal, Switzerland; 4 Laboratory of Biology of Tumor and Development, Groupe Interdisciplinaire de Génoprotéomique Appliqué-Recherche (GIGA-Cancer), University of Liège, Liège, Belgium; 5 Institute of Pathology, University of Bern, Bern, Switzerland; 6 Department of Visceral, Thoracic and Vascular Surgery, Technische Universität Dresden, Dresden, Germany; 7 Department of Gastroenterology, University of Bern, Bern, Switzerland; 8 Department of Surgery, Technische Universität München, München, Germany; 9 Institute of Zoology, Johannes-Gutenberg University, Mainz, Germany; Emory University, United States of America

## Abstract

Meprin-α is a metalloprotease overexpressed in cancer cells, leading to the accumulation of this protease in a subset of colorectal tumors. The impact of increased meprin-α levels on tumor progression is not known. We investigated the effect of this protease on cell migration and angiogenesis *in vitro* and studied the expression of meprin-α mRNA, protein and proteolytic activity in primary tumors at progressive stages and in liver metastases of patients with colorectal cancer, as well as inhibitory activity towards meprin-α in sera of cancer patient as compared to healthy controls. We found that the hepatocyte growth factor (HGF)- induced migratory response of meprin-transfected epithelial cells was increased compared to wild-type cells in the presence of plasminogen, and that the angiogenic response in organ-cultured rat aortic explants was enhanced in the presence of exogenous human meprin-α. In patients, meprin-α mRNA was expressed in colonic adenomas, primary tumors UICC (International Union Against Cancer) stage I, II, III and IV, as well as in liver metastases. In contrast, the corresponding protein accumulated only in primary tumors and liver metastases, but not in adenomas. However, liver metastases lacked meprin-α activity despite increased expression of the corresponding protein, which correlated with inefficient zymogen activation. Sera from cancer patients exhibited reduced meprin-α inhibition compared to healthy controls. In conclusion, meprin-α activity is regulated differently in primary tumors and metastases, leading to high proteolytic activity in primary tumors and low activity in liver metastases. By virtue of its pro-migratory and pro-angiogenic activity, meprin-α may promote tumor progression in colorectal cancer.

## Introduction

Concerted extracellular proteolytic events may promote tumor progression by disrupting physical barriers such as the basement membrane and by processing extracellular matrix, growth factors and cytokines in the tumor stroma. Proteases affect cell adhesion and cell growth as well as individual and collective cell migration [1], [2]. The profound effects of proteases are controlled by the regulation of protease activity at multiple levels including expression levels, activation of zymogen forms, inhibitor levels, and inactivation.

We previously identified meprin-α, a metzincin protease of the astacin family [3], as a new component of the protease network in colorectal cancer [4]. There are two homologous isoforms of meprin: meprin-α, encoded by *MEP1A* on chromosome 6, and meprin-β, encoded by *MEP1B* on chromosome 18 [5]. Meprin-α and meprin-β are co-expressed in small intestine, whereas only meprin-α is expressed in the colon [6]. Meprin-β accumulates at the apical cell surface on enterocytes in the small intestine, whereas meprin-α is secreted unless it is retained at the cell surface through non-covalent association with meprin-β [6]. The single polypeptide chains are not stable, both isoforms form protein complexes of meprin-β dimers, meprin-α and meprin-β tetramers, or multimeric complexes of up to ten meprin-α subunits [7], [8]. Meprin-α is expressed in epithelial cells of the healthy colon mucosa as well as in colorectal cancer [6]. However, in contrast to normal intestinal epithelial cells, which release the protease into the gut lumen, cancer cells secrete the protease in a non-polarized fashion, leading to its accumulation and activation in the tumor stroma [4]. Meprin-α cleaves a range of different substrates *in vitro* [9], including extracellular matrix components of basement membranes [9], [10] but few substrates have been described *in vivo* [11], [12], [13], [14], [15]. Three endogenous meprin inhibitors are described, mannan-binding lectin (MBL) [16], fetuin-A and cystatin C [17].

Meprin-α is secreted from epithelial cells as a zymogen [18]. *In vitro*, the propeptide may be removed using trypsin, yielding the active enzyme. Trypsin thus may activate meprin-α in the gut lumen *in vivo*. An alternative activation system has been identified in co-cultures of the colon carcinoma cell line Caco-2 and intestinal fibroblasts. Fibroblast-derived urokinase-type plasminogen activator (uPA) converts plasminogen into plasmin, which in turn generates active meprin-α [19]. In skin the kallikrein-related peptidase 5 could be identified as a promeprin-α converting enzyme [20].

Here we provide evidence for a pro-angiogenic and pro-migratory activity of meprin-α and investigate the expression, activation and inhibition of this protease in primary tumors, liver metastases and bloodstream in colorectal cancer patients. Our findings show a complex pattern of regulation, which is in accordance with the protease being implicated in the spread of cancer cells from primary sites.

## Results

### Meprin-α promotes cell migration and angiogenesis *in vitro*

**Figure 1:**
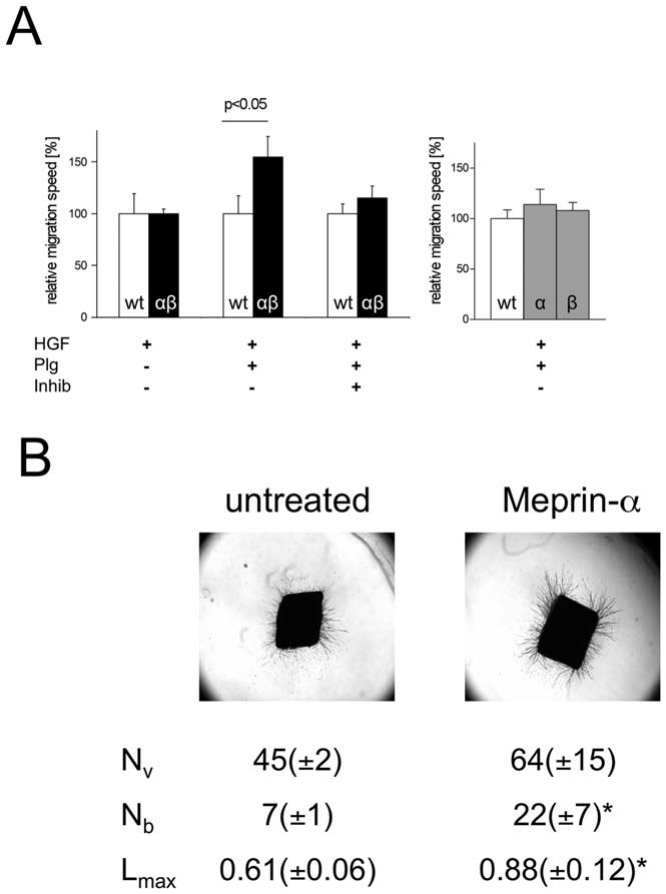
Meprin promotes migration and angiogenesis *in vitro*. (A) Parental MDCK wild-type cells (wt, open columns) and MDCK cells co-transfected with meprin-α and meprin-β (αβ, left panel, filled black columns) or transfected with either meprin-α (α) or meprin-β (β) alone (right panel, filled grey columns) were cultured on laminin 1-coated dishes and exposed to HGF (20 nM) to induce migration in the absence or presence of plasminogen as indicated. HGF-induced migration was tracked for 3–4 h using videomicroscopy. Shown is the average migration speed (+/− SEM) of meprin-transfected cells relative to identically treated wild-type cells. Results are the average from three to four independent experiments for each condition. In the presence of plasminogen (100 nM), HGF-induced migration of meprin-α/β co-transfected cells was significantly enhanced. This effect was reverted in the presence of the meprin inhibitor actinonin (100 nM). The migration of cells transfected with either meprin subunit alone was not different from wild-type cells. (B) Meprin-α promotes angiogenesis. Purified recombinant human active meprin-α was supplemented to the culture medium (0.7 µg/ml) in the rat aortic ring assay and outgrowth of vessels into the collagen gel was quantified using image analysis (see materials and methods). N_v_: number of vessels, N_b_: number of branchings, L_max_: maximum length of vessels. *p<0.05 compared to untreated organ culture. Results are from three independent experiments with triplicates for each condition.

The effect of meprin-α on cell migration was investigated using the well-characterized scattering response of Madin-Darby canine kidney cells (MDCK) cells in response to hepatocyte growth factor (HGF) [21]. The response of meprin-transfected cells and parental MDCK cells was recorded using time-lapse videomicroscopy and quantified as described previously (Fig. 1A) [22]. We compared migration of parental MDCK cells with MDCK cells transfected with either meprin-α alone, meprin-β alone, or co-transfected with both meprin-α and meprin-β. Untreated parental and meprin-transfected MDCK cells do not migrate and grow as cell cluster that eventually form a cell monolayer. A pro-migratory response was induced by adding HGF. Meprin-transfected and control MDCK cells migrated similarly in the presence of HGF alone. Only after adding plasminogen as a source to generate plasmin, which in turn activates meprin-α [19], the migration speed of meprin-α/β co-transfected cells was increased by approximately 50% as compared to wild-type cells. This difference was abolished by the addition of the meprin inhibitor actinonin. Notably, tethering of meprin-α to the cell surface through meprin-β is essential to enhance this pro-migratory response in the presence of HGF and plasminogen, as MDCK cells transfected with either meprin-α or meprin-β alone migrated similarly to parental cells.

Purified recombinant active human meprin-α promoted the angiogenic response in the rat aortic ring assay [23] (Fig. 1B). Computer-assisted image analysis of rat aortic explants in 3-dimensional collagen gel culture indicated that meprin-α enhanced the outgrowth of capillaries compared to control cultures in terms of both the length (increase by 44%, p<0.05) and the number of branchings (3-fold increase, p<0.05).

### Meprin-α expression in colorectal cancer at progressive tumor stages

**Figure 2:**
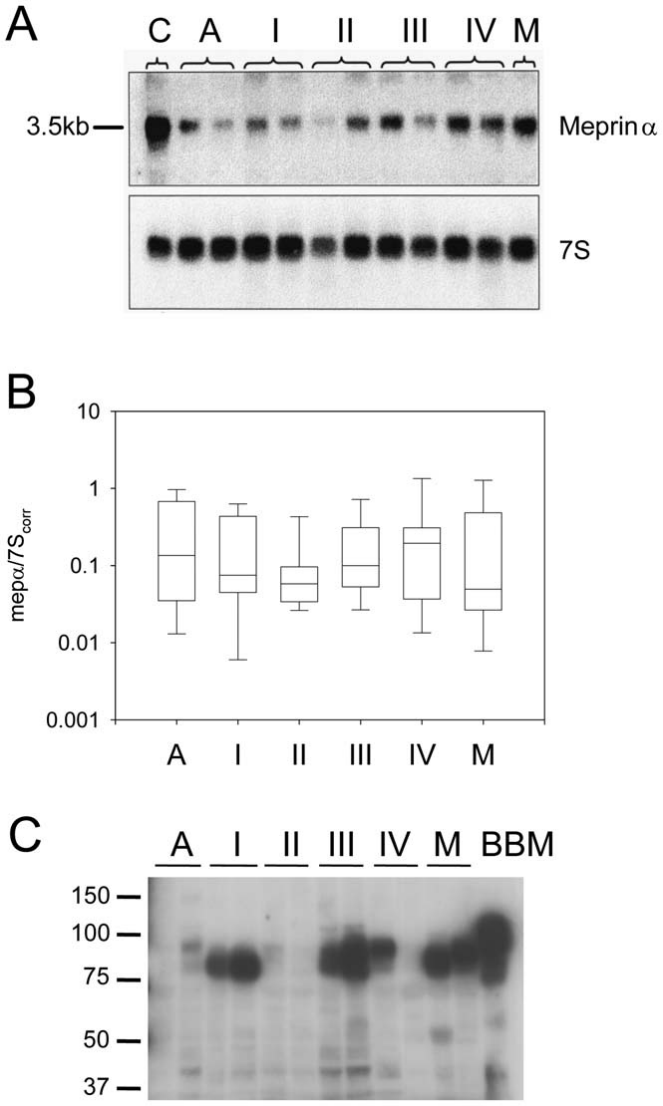
Expression of meprin-α mRNA and protein in adenomas, primary tumors I to IV and in liver metastases. (A) Representative Northern blot for meprin-α on total RNA (12 µg) extracted from tumor samples of patients with different tumor stages (A adenoma, primary tumors stages UICC I to UICC IV, M liver metastases). Normal control RNA from healthy colon mucosa is also shown (lane C). Varying levels of a single 3.5 kb mRNA species were detected. The amount of loaded RNA was assessed by reprobing the blot for 7S RNA. (B) Quantification of meprin-α mRNA levels relative to 7S RNA. Box plot representation of densitometric values obtained from Northern blots. Box includes lower quartile, median and upper quartile, whiskers indicate 5–95 percentile range (N = 12 in each group). No significant differences between different groups. (C) Representative Western blot for meprin-α on protein extracts from tumor samples (40 µg). As a control, purified human brush border membranes from small intestine (20 µg) were also analyzed (BBM), where meprin-α is associated with transmembrane meprin-β in heterodimeric protein complexes. Meprin-α was detected in a subset of the tumors. In order to reveal weak signals, the membrane was overexposed with respect to tumor samples that exhibited strong expression.

**Figure 3:**
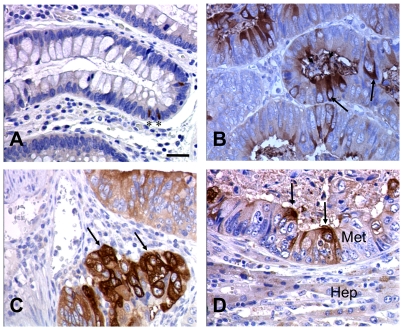
Meprin-α is expressed in cancer cells. Immunostaining for meprin-α on tissue sections from formalin-fixed and paraffin-embedded colorectal tissue samples using a rabbit polyclonal primary antibody together with a peroxidase-coupled secondary antibody and diaminobenzidine as chromogenic substrate. Counter stain: hematoxylin. Objective magnification: 40×. Bar = 50 µm. (A) In normal colon mucosa meprin-α is barely detectable. Occasionally, signals were detected at the base of crypt regions in the cytosol of cells with apically localized nuclei (asterisks). The specificity of these signals and the identity of the cells was not further investigated. (B) Representative sample of a meprin-α positive primary tumor stage III. Tumors exhibit a mosaic expression pattern. In cancer cell clusters, cells with strong signals for meprin-α (arrows) are intermixed with cells that show weak signals or no signal at all. Cells in the stroma do not express meprin-α (C) Representative sample of meprin-α positive primary tumor stage IV showing clusters of meprin-α positive cancer cells (arrows). (D) Cancer cells express meprin-α (arrows) in liver metastases (Met). The surrounding normal liver tissue (Hep) is only weakly stained.

**Figure 4:**
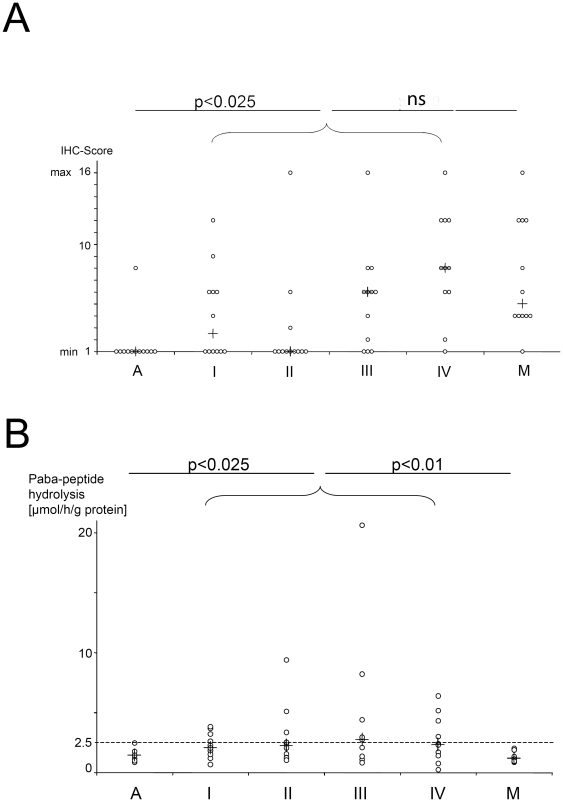
Meprin-α protein and activity in primary tumors and in liver metastases. (A) Semi-quantitative assessment of meprin-α expression in colorectal carcinomas using an immunohistochemical score (minimum score 1, maximum score 16). + = median values. (B) Increased proteolytic activity of meprin-α in primary tumors stages I to IV as compared to adenomas and metastases. Proteolytic activity of meprin-α was measured using the artificial substrate N-benzoyl-L-tyrosyl-p-amino benzoic acid (paba-peptide). The threshold value of 2.5 µmol/h/g protein corresponds to two standard deviations above the mean value in the adenoma group. An increase of proteolytic activities above threshold was found in 36%, 40%, 44% and 33% of the primary tumor samples at stages UICC I, II, III and IV, respectively, but not in liver metastases. Statistical analysis was performed between primary tumors stages I to UICC IV as one group and adenomas, or metastases (one-sided Wilcoxon rank sum test of independent samples). + = median values.

Variable levels of a single 3.5-kb meprin-α mRNA were detected in patient samples at all stages including adenomas, which did not correlate significantly with tumor stages (Fig. 2A,B). Alternative splicing of meprin-β in cancer has been previously reported [24]. No evidence for an alternatively spliced mRNA isoform of meprin-α was found in tumors. On immunoblots meprin-α protein was only weakly detectable or absent in adenomas, whereas a subset of cancer samples, including primary tumor stages I to IV and liver metastases, contained high amounts of meprin-α protein (Fig. 2C). Purified brush border membranes from human small intestine, which contain both meprin-α and meprin-β, were analyzed as a positive control. As described previously [4], the molecular species of meprin-α in tumors were 10 to 25 kDa smaller than the predominant 100-kDa protein from brush border membranes. Immunostaining for meprin-α in primary tumors and liver metastases revealed clusters of cancer cells with strong signals adjacent to negative cancer cells (Fig. 3). All adenomas but one scored the minimum value 1 (Fig. 4A), whereas primary tumors and liver metastases scored higher (p<0.025 for primary tumors stages I to IV compared to liver metastases and adenomas).

### Meprin-α activity, zymogen activation and inhibition

Meprin-α activity was specifically measured in protein extracts from the tissue samples in the presence of a broad range protease inhibitor targeting all protease classes except metalloproteases. Meprin-α activity was low in adenomas, whereas significantly enhanced activity was measured in a subset (33 to 44%) of primary tumors of stages I to IV (Fig. 4B, p<0.01 for stages I to IV compared to adenomas). In liver metastases, unexpectedly, meprin-α activity levels were as low as those in adenomas, despite significant expression of the protein (p<0.01 compared to primary tumors stages I to IV).

**Figure 5:**
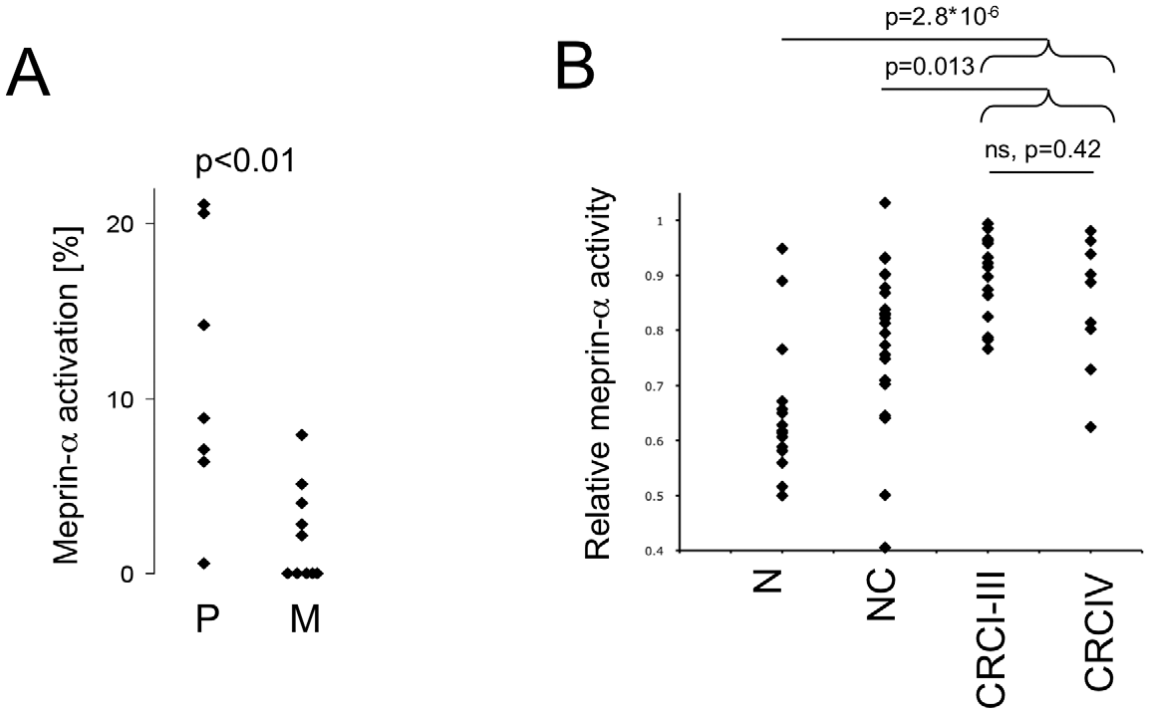
Meprin-α zymogen activation and inhibition in colorectal cancer. Meprin-α was immunoprecipitated from tumor samples, divided into two aliquots to derive endogenous and total activity, as described in Materials and Methods. (A) Ratio of endogenous meprin-α activity versus total activity in primary tumors (P, N = 7) and liver metastases (M, N = 10). Zymogen activation is significantly enhanced in primary tumors compared to liver metastases. (B) Serum samples from colorectal cancer patients exhibit lower inhibitory activities against meprin-α. Proteolytic activity of purified human recombinant meprin-α (800 ng/ml) was assayed in the presence of 20% human serum. Activities are shown relative to the reference assay with of 20% phosphate-buffered saline pH 7.3 ( = 1). N: Normal healthy controls (N = 19), NC: Non-cancer patients group, which includes samples from 22 inviduals with inflammatory disorders of the intestine, CRCI–III: colorectal cancer patients (N = 15) stages I–III. CRCIV: colorectal cancer patients (N = 9) stage IV. Significantly reduced inhibition of meprin-α in serum from colorectal cancer patients.

The apparent discrepancy between low activity levels and high meprin-α protein levels in liver metastases (Fig. 2C and 3) might be due to lack of meprin-α zymogen activation, or an inhibitor specifically present in liver metastases. To determine the extent of zymogen activation in tumor samples we first immunoprecipitated zymogen and active forms of meprin-α and measured activity directly on the beads. This value, which represented the endogenously activated protease pool, was compared to the activity in immunoprecipitates after maximum zymogen activation by limited trypsin treatment [18]. The zymogen form of meprin-α predominated in all samples. However, endogenous activation of meprin-α was significantly higher in primary tumors than liver metastases (Fig. 5A). In primary tumors I–IV, as much as 20% of total meprin-α was activated endogenously, and all but one sample displayed endogenous activation above 5%. Conversely, endogenous activation was below 5% in all but two samples from liver metastases. In conclusion, increased zymogen activation may contribute to the increased meprin-α activity at primary tumor sites as compared to metastatic sites in liver.

### Reduced inhibition of meprin-α in sera from colorectal cancer patients

Circulating cancer cells might encounter factors in the blood that modulate meprin-α activity. Indeed, an activity assay of recombinant meprin-α in the presence of 20% human sera from healthy controls and a group of patients with intestinal inflammatory disorders indicated inhibition by 35% (range 5–50%) and 22% (range 0–59%), respectively. In contrast, sera from colorectal cancer patients (stages I–IV) inhibited meprin-α at a significantly lesser extent (12% on average, 0–37%, p = 2.8*10^−6^ compared to healthy controls, p = 0.013 compared to patients with inflammatory disorders) (Fig. 5B). Sera from patients at stages I–III showed a reduced inhibitory capacity compared to normal patients that was not significantly different from patients at stage IV. Inhibition in serum was independent of MBL, as MBL serum concentrations did not differ significantly between the study groups and also did not correlate with meprin-α inhibition (not shown). In addition, recombinant MBL did not inhibit human, mouse and rat meprin in the N-benzoyl-L-tyrosyl-p-amino benzoic acid (paba-peptide) assay (Table S1).

In summary, there is a dynamic and complex regulation pattern for the activity of meprin-α in colorectal cancer. Increased zymogen activation may contribute to higher proteolytic activity in primary tumors stages I to IV, and the inhibitory activity against meprin-α is reduced in the blood in colorectal cancer patients.

## Discussion

This study implies that meprin-α is active in the transition from benign growth (adenomas) to malignant primary tumors. Paba-peptide activity in the cancer tissue is specific for meprin in our assay, as we took care to suppress any unspecific activity by supplementing the assay with a broad range protease inhibitor cocktail. Notably, chymotrypsin-like proteases, which are the only known enzymes able to cleave paba-peptide apart from meprin [25], are inhibited under these conditions. Meprin-α protein and activity increased in the latter group even though there was no significant difference of corresponding mRNA levels between different groups. Adenomas by definition are characterized by an increased growth rate of cells with normal differentiation and cell polarization [26]. Therefore, akin to the normal colon mucosa, meprin-α may be secreted and subsequently lost into the gut lumen, whereas the protease is retained within the tumor tissue in colorectal cancer due to aberrant non-polarized secretion, a mechanism described by us previously [4]. In addition there is evidence for a posttranscriptional regulation of meprin-α from a previous study, as meprin-α mRNA was detectable by *in situ* hybridization in both crypt and villus regions, whereas the protein as detected by immunostaining was only present in villus enterocytes [6].

In colorectal cancer, primary tumors contained increased meprin-α activity and frequently harbored a subpopulation of cancer cells with strong meprin-α protein expression, in particular at UICC stages III and IV, i.e. after progression to metastasis to lymph nodes and distant sites (Fig. 2 and 3). The dissemination of cancer cells thus correlates with increased meprin-α protein and activity. However, we note that the correlation between immunoblot signals, immunostaining scores and meprin-α-activity levels in the groups of primary tumors is not tight. The immunostaining score does consider frequency and intensity of meprin-α signals among cancer cells only within a locally restricted area of the sample, whereas the immunoblot signal is an average of the whole sample including secreted meprin-α accumulating in the tumor stroma [4]. The meprin-α activity level is additionally affected by zymogen activation and the presence of inhibitors.

Sera from patients with colorectal cancer displayed significantly lower inhibitory activity towards meprin-α than healthy controls and patients with intestinal inflammatory diseases, indicating that emigration of circulating meprin-α expressing cancer cells out of blood vessels may be facilitated. The inhibitory activity in serum was not due to mannan-binding lectin, an endogenous meprin inhibitor reported previously [16], and thus is contributed by one or several additional inhibitor(s). In a recent report, fetuin-A and cystatin C have been reported as such endogenous meprin inhibitors [17]. In fact, using recombinant human MBL, we found no inhibition of meprin (Table S1). This finding is in contrast to the report by Hirano et al. [16]. However, our data are in accordance with the lack of a correlation between inhibition by patient sera and MBL concentration. Secondly, as Hirano et al. used MBL purified from human serum for their meprin inhibition assays, we speculate that the discrepancy between our and their data is due to contaminating factors from the human serum, such as fetuin-A and/or Cystatin C.

There is a dynamic and complex regulation pattern of the activity of meprin-α in colorectal cancer. Increased zymogen activation contributes to higher proteolytic activity in primary tumors stages I to IV (Fig. 5A), and the inhibitory activity against meprin-α is reduced in the blood in colorectal cancer patients (Fig. 5B). The inefficient activation in liver metastases is in accordance with the previous observation that the uPA-system, a meprin-α activator, is inactive in liver metastases, due to the lack of uPA activation and the over-expression of plasminogen activator inhibitor [27], [28]. Even though many matrix metalloproteases (MMPs) are known to be increased in invasive primary tumors in colorectal cancer [29], there are few analyses that include liver metastases. In one such study, MMP-9 was shown to be increased in primary tumors but not liver metastases [30]. That study and our data point to significant differences between protease networks in primary tumors and liver metastases, which is relevant for therapeutic approaches using protease inhibitors.

We studied cell migration in MDCK cells expressing meprin-α at the cell surface bound in heterodimeric protein complexes with transmembrane meprin-β [31]. Meprin-expressing cells migrated significantly faster on laminin-1 coated dishes in the presence of plasminogen and HGF. Meprin-α might be activated at the cell surface by plasmin, generated from plasminogen through the activity of urokinase-type plasminogen activator (uPA) bound to its receptor (u-PAR), which both are known to be up-regulated by HGF in MDCK cells [32]. This system most probably also contributes to the activation of meprin-α in tumors *in vivo*, as u-PA and u-PAR are up-regulated in colorectal cancer [33], [34], [35], [36]. In our migration assay, the pro-migratory effect is indeed due to meprin-α, as plasmin activates meprin-α but not meprin-β [7] and because actinonin, which reverses the pro-migratory effect, does inhibit meprin-α with approximately 100-fold higher potency (K_i_ 2*10^−8^ M) than meprin-β (K_i_ 2*10^−6^ M) [10].

Both laminin-1 and laminin-5 are cleaved by meprin-α *in vitro* [37], and we speculate that meprin-α might expose cryptic pro-migratory epitopes in a similar fashion as has been previously shown with laminin-5 and laminin-1 for MT1-MMP [38] and elastase [39], respectively. The uPA-plasminogen system is also crucially important in angiogenesis [23], [40], and plasmin in turn might activate meprin-α as a downstream angiogenic effector in colorectal cancer. In line with our data, a pro-angiogenic effect of meprin-α has also been observed in a morpholino knockdown in zebrafish [14].

The pro-angiogenetic activity was evident with extracellular soluble meprin-α, whereas its pro-migratory effect was evident on cells with meprin-α at their cell surface. Therefore, the pro-angiogenetic effect of meprin-α released by cancer cells may be promoted by its proteolytic activity outside and distant from pro-angiogenic/vasculogenic cells. Secreted meprin-α may thus condition the tumor environment to promote enhanced angiogenesis together with other pro-angiogenic factors, whereas meprin-α tethered at the cell surface may promote the migration of cancer cells themselves. Both meprin-dependent processes are expected to jointly promote tumor progression.

In conclusion, this study reveals a complex and dynamic regulation pattern for meprin-α in tumor progression. The transition to malignant stages in colorectal tumors correlates with increased meprinα activity at primary tumor sites, consistent with a role in invasion and metastatic dissemination of cancer cells. A role for meprin-α in this process is further supported by its pro-angiogenic and pro-migratory activity. Our data also show that the promotion of migration depends on the simultanous expression of meprin-β tethering meprin-α onto the cell surface. The lack of detectable meprin-β mRNA in the whole tumor does not exclude its induction in a subpopulation of cancer cells. Our data are in agreement with the view that the meprin-α/β co-expressing cancer cells are likely to migrate away from the tumor mass. We previously described that meprin-β weakens intercellular junctions [41]. Future studies should focus on the analysis of the role of meprins in emigration of these cancer cells. The inhibitory activity towards meprin-α is lower in sera from patients with colorectal cancer, which might facilitate dissemination of meprin-expressing cancer cells. Therapeutic approaches with protease inhibitors targeting meprin-α in primary tumors and in the bloodstream might counteract tumor progression. On the other hand, meprin-α activity is low in liver metastases, and therefore inhibition of this protease at this site is not expected to be an effective treatment.

## Materials and Methods

### Recombinant human meprin

Recombinant active meprin-α and meprin-β was purified from insect cells as described previously [7], [42]. No residual trypsin activity was detectable in the purified recombinant meprins.

### Cell migration assay

Madin-Darby canine kidney (MDCK) cells were grown in minimum essential medium (MEM) supplemented with 5% FCS, 100 U/ml of penicillin and 100 µg/ml of streptomycin (cell culture media and supplements from Invitrogen, Basel, Switzerland). Meprin-transfected MDCK cells [31] and wild-type parental MDCK cells were seeded in laminin-1 coated 12-well plates at a density of 10'000 cells/well (12-well plates from Costar, Cambridge, MA, U. S. A., purified laminin-1 from Engelbreth-Holm-Swarm mouse sarcoma, Sigma, St. Louis, MO, U. S. A) and incubated overnight in MEM with 5% FCS. After a preincubation of 48 h in serum-free medium (Advanced DMEM, Invitrogen) containing 20 nM hepatocyte growth factor (HGF), migrating cells were recorded using time lapse videomicroscopy for 3–4 h. Plasminogen (American Diagnostica, Pfungstadt, Germany) and actinonin (Sigma) both were added at 100 nM. Migration tracks were evaluated as reported previously [22]. HGF induced migration of MDCK cells at speeds between 27 and 84 µm/h. In the absence of HGF, MDCK cells grow in clusters and do not migrate. Due to the technical limitation of the experimental setup used, multiple migration experiments had to be carried out sequentially on different days. As the average migration speeds varied substantially between experimental sessions irrespective of the conditions assessed. the measurements were normalized to the migration of wild-type cells observed in each experimental session.

### Angiogenesis assay (aortic ring assay)

Rings of rat thoracic aorta (1 mm length) were cultured in 3-dimensional collagen gels (rat tail interstitial collagen gel, 1.5 mg/ml, Serva, Heidelberg, Germany) [23]. Cultures were maintained for 9 days at 37°C in 6 ml MCDB 131 (Invitrogen) with 25 mM NaHCO3, 1% glutamine, 100 U/ml penicillin, and 100 µg/ml streptomycin in the presence or absence of 0.7 µg/ml purified recombinant active human meprin-α. The angiogenic response was quantified using image analysis with the software Aphelion 3.2 (Adcis) on three independent experiments (each in triplicate). Following geometrical and morphological parameters were determined: number of microvessels (Nv), maximal microvessel length (Lmax) and total number of branches in microvessels (Nb) [43].

### Patients

Collection of tumor specimens during surgical interventions was approved by the Ethical Committee of the Medical Faculty, University of Bern, Switzerland. Written informed consent was obtained from all patients. Carcinomas were staged according to UICC nomenclature. The study included 72 patients (49/23 male/female, median 64.5 yrs, range 20–90 yrs), with 12 patients in each of the following six groups: Adenomas (5/7 m/f, median 68.5 yrs, range 20–89 yrs), primary tumors stage I (7/5 m/f, median 72.5 yrs, range 60–90 yrs), stage II (7/5 m/f, median 73 yrs, range 50–82 yrs), stage III (10/2 male/female, median 64 yrs, range 48–84 yrs), stage IV (9/3 m/f, median 60.5 yrs, range 43–84 yrs) and liver metastases (11/1 m/f, median 57.5 yrs, range 31–84 yrs). The samples of this study group have been analyzed by Northern and Western blotting, immunohistochemistry and subjected to meprin activity assays. Experiments shown in Fig. 5A/B included additional eight samples from liver metastases (6/2 m/f, median 68 yrs, range 37–82 yrs). MBL concentrations and meprin-α inhibition were assayed in sera from 19 healthy controls (6/13 m/f, range 26–49 yrs), 22 non-cancer patients (8/14 m/f, range 22–76 yrs; 1 celiac disease, 2 ulcerative colitis and 19 Crohn's disease patients) and 24 colorectal cancer patients (17/7 m/f, range 50–76 yrs).

### Northern blots

^32^P-labeled RNA-probes were synthesized from 600 ng linearized plasmids (pBluescript II KS(+); Stratagene, La Jolla, CA, U. S. A.) containing human meprin-α or 7S RNA sequences using T3 and T7 RNA polymerases (Roche Diagnostics, Rotkreuz, Switzerland) in the presence of 50 µCi [^32^P]-CTP (NEN, Boston, MA, U. S. A.), as described previously [4], [44]. Northern blots of total RNA (12 µg) from normal colon and carcinoma tissue specimens on a nylon membranes (Genescreen; NEN) were hybridized sequentially with ^32^P-labeled human meprin-α specific and 7S RNA-specific single-stranded RNA probes [4]. Hybridization signals on X-ray films were quantified using densitometry with background correction. Meprin-α mRNA levels were normalized relative to 7S RNA.

### Tissue protein extracts

Using a Teflon homogenizer, frozen tissue pieces were disrupted in 5 ml ice-cold 25 mM Tris-HCl, pH 8.0, 50 mM NaCl, supplemented with protease inhibitors (Complete EDTA-free, Roche Diagnostics). Homogenates were lysed in the presence of 1% deoxycholic acid (Sigma-Aldrich, St. Louis, MO, U. S. A.) and 1% NP-40 (Sigma-Aldrich) on ice for 1 h. Protein content was measured using the bicinchoninic acid (BCA) protein assay (Pierce Biotechnology Inc., Rockford, IL, U. S. A.).

### Immunoblots

40 µg protein extract per sample were separated using SDS-PAGE (7.5%) under reducing conditions and blotted onto a polyvinylidene difluoride (PVDF)-membrane (Millipore, Bedford, MA, U. S. A.). Subsequent incubations were done in 20 mM Tris-HCl, pH 7.5, 138 mM NaCl. Blocked membranes (5% dry milk) were probed with meprin-α specific polyclonal antibodies directed towards the TRAF-domain of meprin-α (1∶500 in 1% dry milk) [6] and a peroxidase-coupled anti-rabbit secondary antibody (from Amersham Biosciences, Buckinghamshire, UK; used 1∶10,000 in 1% dry milk). Meprin-α-specific bands were visualized with enhanced chemiluminescence (ECL plus, Amersham Biosciences). Equal loading and integrity of the proteins after Western blotting was confirmed by Coomassie-staining of total protein after immunoblotting (Fig. S1).

### Meprin-α proteolytic activity and inhibition assays

Meprin-α activity was measured with N-benzoyl-L-tyrosyl-p-amino benzoic acid (paba-peptide) as substrate. Chymotrypsin-like proteases are the only known enzymes able to cleave paba-peptide apart from meprin. 100 µl protein extract (1 mg/ml) was incubated with 100 µl of 40 mM paba-peptide (Bachem AG, Bubendorf, Switzerland) in 50 mM Tris-HCl, pH 7.5, 1 mM MgCl2 and incubated for 6 h at 37°C. To inhibit unspecific paba-peptide activity in the protein extract, all proteases except metalloproteases were inhibited by the broad range protease inhibitor Complete Protease Inhibitor Cocktail (Roche Diagnostics). Released p-amino benzoic acid was determined using the colorimetric assay according to Bratton-Marshall and quantified against a p-amino benzoic acid standard [45].

Inhibition assays were performed in the presence of the broad range protease inhibitor Complete Protease Inhibitor Cocktail (Roche Diagnostics) with 1) human recombinant meprin-α and meprin-β (purified from an Insect Baculovirus system), 2) with mouse meprin-α and rat meprin-β (purified from transfected mammalian cells, gift from Judith Bond), 3) with cell lysates from meprin-α/β co-transfected MDCK cells and 4) with human purified intestinal brush border membranes in the presence of recombinant human MBL (purified from transfected mammalian cells, from Sino Biologicals, Beijing, China). Substrates used in the inhibition assay were either the fluorogenic substrate Mca-YVADAPK(Dnp)-OH (at 10 nM to 2 µM, from R+D Systems, Minneapolis, MN U. S. A.) or azocasein (at 10 mg/ml, from Sigma-Aldrich, St. Louis MO, U. S. A.). The small inhibitors actinonin (100 µM) and EDTA (10 mM), known to inhibit meprin, were used as control.

### Immunoprecipitation

1 ml of protein extract from primary tumors and liver metastases (1–5 mg/ml) was incubated with anti-human meprin-α monoclonal antibody (gift of Judy Bond, Hershey, PA, U. S. A.) and immune complexes were captured on protein-A sepharose beads (Amersham Biosciences, Buckinghamshire, UK). Beads with bound immune complexes were washed three times with isotonic phosphate-buffered saline (pH 7.3), 0.5% NP-40 (Sigma-Aldrich), 0.05% deoxycholic acid (Sigma-Aldrich), and twice with 125 mM Tris-HCl, pH 8.2, 500 mM NaCl, 1 mM EDTA, 0.5% NP-40. To measure endogenous and total meprin-α activities, immunoprecipitates were divided into two equal parts, resuspended in 100 µl isotonic phosphate-buffered saline (pH 7.3), and one part was incubated for 2 h at 37°C with 20 µg/ml trypsin to activate the zymogen form. Meprin-α activities were then measured in the presence of 50 µg/ml soybean trypsin inhibitor (Roche Diagnostics).

### Quantification of mannan-binding lectin (MBL)

Oligomeric MBL in patient sera (diluted 1∶100) and tumor protein extracts (1 mg/ml protein diluted 1∶10) was measured using the MBL Oligomer ELISA Kit (AntibodyShop, Gentofte, Denmark).

### Immunohistochemistry

2-µm paraffin sections were dewaxed, rehydrated, microwaved (5 min in 10 mM sodium citrate, pH 6.0) and incubated sequentially with meprin-α specific rabbit antisera [6], biotinylated anti-rabbit antibodies and avidin-biotin-peroxidase complex (Vectastain ABC-Immunostaining Kit, Vector Laboratories, Burlingame, CA, U. S. A.) in 25 mM Tris-HCl, pH 7.5, 140 mM NaCl, and diaminobenzidine (Immuno Pure Metal Enhanced DAB; Pierce Chemical Co., Rockford, IL, U. S. A.) as chromogenic substrate. Signals were analyzed semi-quantitatively applying a score (range 1–16) considering the frequency and staining intensity of meprin-α positive cancer cells.

### Statistical tests

Criteria for normal distribution were not fulfilled in our clinical study groups, and Wilcoxon rank sum tests (unpaired samples) were applied. Differences between endothelial cell migration in the aortic ring assay, as well as between migration of meprin-transfected and wild-type MDCK cells were assessed using the Student's t-test for paired data. Statistical calculations and graphical representations were created using Sigmaplot 9 and the statistical work package R (http://www.r-project.org/).

## Supporting Information

Figure S1 **Coomassie-stained immunoblot of protein lysates from tumor samples.** 40 µg protein extract per sample were separated using SDS-PAGE (7.5%) under reducing conditions and blotted onto a polyvinylidene difluoride (PVDF)-membrane. The Coomassie-stained blot confirms equal loading and blotting of proteins across the samples.
(JPG)

Table S1 **Inhibition assay of Mannan-binding lectin (MBL) and meprin-α and meprin-β.** There was no evidence for an inhibitory activity of Mannan-binding lectin towards recombinant human, mouse and rat meprins, and human meprins in transfected cells and in purified human intestinal brush border membrane.
(PDF)
