# Supplementary figures and images for: Enhanced Activity of Meprin-α, a Pro-Migratory and Pro-Angiogenic Protease, in Colorectal Cancer
Source: PLoS One. 2011 Nov 11;6(11):e26450. doi: 10.1371/journal.pone.0026450 (PMC3214016; doi:10.1371/journal.pone.0026450)

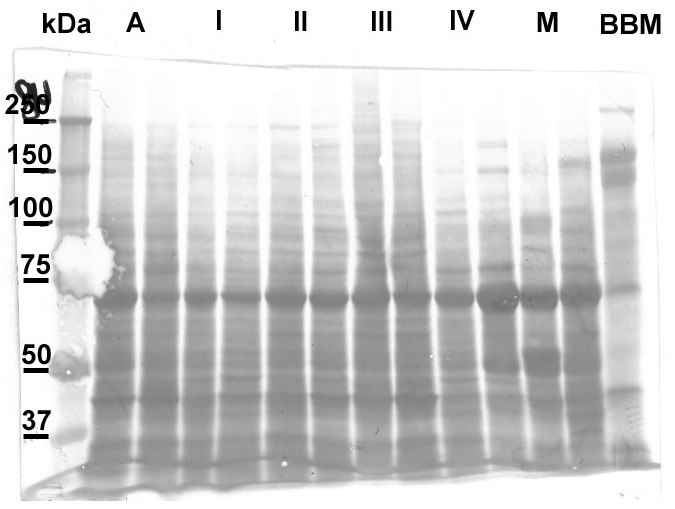

Supplement: Figure S1 — Coomassie-stained immunoblot of protein lysates from tumor samples. 40 µg protein extract per sample were separated using SDS-PAGE (7.5%) under reducing conditions and blotted onto a polyvinylidene difluoride (PVDF)-membrane. The Coomassie-stained blot confirms equal loading and blotting of proteins across the samples. (JPG) [file pone.0026450.s001.jpg]
