# Supplementary material for: Enhanced Activity of Meprin-α, a Pro-Migratory and Pro-Angiogenic Protease, in Colorectal Cancer
Source: PLoS One. 2011 Nov 11;6(11):e26450. doi: 10.1371/journal.pone.0026450 (PMC3214016; doi:10.1371/journal.pone.0026450)
Supplement: Table S1 — Inhibition assay of Mannan-binding lectin (MBL) and meprin-α and meprin-β. There was no evidence for an inhibitory activity of Mannan-binding lectin towards recombinant human, mouse and rat meprins, and human meprins in transfected cells and in purified human intestinal brush border membrane. (PDF) [file pone.0026450.s002.pdf]

Table S1: Mannan-Binding Lectin does not inhibit meprin

**source of meprin**	**species**	**[meprin]**	**substrate**	**[MBL[3]]**	**molar ratio I/E[5]**	**relative activity ± SD**
recombinant meprin α	human	1nM	Fluorogenic	1μM	$1x10^3$	116.23 ± 3.84
recombinant meprin α	human	1nM	Fluorogenic	200nM	200	91.34 ± 5.56
recombinant meprin α	human	1nM	Fluorogenic	50nM	50	95.67 ± 6.22
recombinant meprin α	human	1nM	Fluorogenic	10nM	10	129.00 ± 14.81
recombinant meprin β	human	0.5nM	Fluorogenic	1μM	$2x10^3$	75.30 ± 2.98
recombinant meprin β	human	0.5nM	Fluorogenic	200nM	400	66.24 ± 9.98
recombinant meprin β	human	0.5nM	Fluorogenic	50nM	100	66.67 ± 1.54
recombinant meprin β	human	0.5nM	Fluorogenic	10nM	20	75.38 ± 2.70
BBM	human	NA[6]	Fluorogenic	2μM	NA	93.38 ± 4.12
MDCKα/β cells	human	NA	Fluorogenic	2μM	NA	89.83 ± 5.44
recombinant meprin α	mouse	5nM	Fluorogenic	2μM	400	134.88 ± 0.59
recombinant meprin α	mouse	5nM	Fluorogenic	1μM	200	142.10 ± 2.31
recombinant meprin α	mouse	5nM	Fluorogenic	0.5μM	100	138.67 ± 3.24
recombinant meprin β	rat	0.5nM	Fluorogenic	2μM	4000	116.31 ± 3.97
recombinant meprin β	rat	0.5nM	Fluorogenic	1μM	2000	116.45 ± 0.54
recombinant meprin β	rat	0.5nM	Fluorogenic	0.5μM	1000	107.48 ± 2.38
recombinant meprin α	mouse	1μM	Azocasein	10μM	10	104.12 ± 3.51
recombinant meprin β	rat	1μM	Azocasein	10μM	10	105.08 ± 2.10

[1] purified human intestinal brush border membranes

[2] MDCK cells co-transfected stably with human meprin α and meprin β cDNAs

[3] recombinant Mannan-Binding Lectin

[4] fluorogenic peptide substrate: Mca-YVADAPK(Dnp)-OH

[5] ratio inhibitor / enzyme

[6] not applicable
